# Internationalization of scientific publishing in a multipolar world

**DOI:** 10.1590/0102-311XEN067325

**Published:** 2025-05-23

**Authors:** Marilia Sá Carvalho, Luciana Dias de Lima, Luciana Correia Alves

**Affiliations:** 1 Programa de Computação Científica, Fundação Oswaldo Cruz, Rio de Janeiro, Brasil.; 2 Escola Nacional de Saúde Pública Sergio Arouca, Fundação Oswaldo Cruz, Rio de Janeiro, Brasil.; 3 Instituto de Filosofia e Ciências Humanas, Universidade Estadual de Campinas, Campinas, Brasil.

The internationalization of Brazilian journals has been widely debated and is seen as an important element for strengthening national science and integrating it into the global academic discourse. Overall, internationalization refers to the process of integrating journals, researchers, and institutions into global circuits of scientific production, dissemination, and evaluation. This involves collaboration among authors from different countries, inclusion in international databases, and adherence to quality criteria established by global organizations.

However, although it is often presented as a pathway to promote visibility and necessary exchanges, the debate on internationalization carries a series of contradictions - especially when viewed from the perspective of countries with lower participation in the traditional circuits of international science exchange. Thus, despite the substantial progress Brazil has made in recent years, it remains a challenge for the country to place its journals and publications among world’s major periodicals.

From a more pragmatic perspective, some authors highlight the obstacles and possible strategies for increasing the international impact of Brazilian publications ^1^ ^,^ ^2^. The language barrier faced by researchers from non-Anglophone countries is among such obstacles ^3^. The Scientific Electronic Library Online (SciELO), the large open access digital library that hosts scientific journals from various countries, supports the publication of papers in English as indispensable for increasing citations and impact indicators, thus enhancing the recognition and visibility of research and its authors in the international community ^4^. The low geographical diversity of the composition of editorial boards, authors, and reviewers is also an aspect that challenges Brazilian journals. Many journals’ editorial boards are predominantly national, which hinders international integration and external recognition. Authors from central countries, in turn, only publish in Brazil when their articles are coauthored with local or Latin American researchers.

Another aspect of this debate questions what actually meant by internationalization of science and scientific journals ^5^. CSP has addressed this issue in other forums ^6^, but it is important to remember that only six conglomerates control the scientific publishing market, with astonishing profit margins ^7^. Open access, coupled with internationalization, is enabled via fees charged to authors, funding agencies, and institutions. It is estimated that scientists have spent over USD 1 billion in four years to publish in open access in these venues. Therefore, concentration and high cost hinder the inclusion of journals and researchers from countries with fewer resources, impairing a more equitable and inclusive internationalization.

The CAPES Journal Portal (https://www.periodicos.capes.gov.br/index.php/sobre/nossa-historia.html), created in 2000, provided access to articles from more than 50,000 scientific journals for about 500 Brazilian institutions, ensuring access to knowledge produced abroad, mainly in developed countries. More recently, CAPES (Brazilian Coordination for the Improvement of Higher Education Personnel) signed transformative agreements with commercial publishers, ensuring the payment of Article Processing Charges (APCs) (https://www.periodicos.capes.gov.br/index.php/acessoaberto/acordos-transformativos.html). Such agreement aims to facilitate the transition from subscription-based access to an open access model, thereby promoting greater visibility to national scientific and technological production. Moreover, publication in these journals will no longer be exclusive to researchers with well-funded projects, thus democratizing publication. Although paying APCs to publish abroad aims to internationalize Brazilian science, it also transfers significant amounts of resources out of the country. In 2025, the CAPES contract with the publisher Wiley cost USD 8.3 million (data obtained via the Freedom of Information Act, protocol 23546.018379/2025-17). By comparison, the CNPq/CAPES call for proposals in 2024 allocated only BRL 6 million to support 272 Brazilian journals, an average of BRL 22,000 per journal.

The transformative agreements combine a receptive internationalization - in which one reads what is produced abroad - and an export-oriented approach - which seeks to disseminate locally produced science. Both processes are inherent to a unipolar world in which the Global South is, by definition, subordinate ^8^. The so-called Global South comprises countries as diverse as Brazil, India, South Africa, Mexico, Nigeria, Argentina, Indonesia, and others from Latin America, Africa, and parts of Asia. It is a geopolitical and epistemological category that encompasses nations historically marginalized in the international flows of science, technology, and innovation. In contrast, the Global North includes countries such as the United States, the United Kingdom, Germany, France, Canada, and Japan, among others, which concentrate the main research centers, publish most high-impact journals, and set the parameters for scientific excellence on a global scale.

In this context, internationalization is often portrayed as a one-way street, marked by the subordination of knowledge produced in the Global South. This subordination is not solely economic or technological; it manifests in the uneven validation of knowledge, the imposition of publication norms, and the implicit notion that science produced in central countries is universal, whereas that produced in the periphery is local, contextual, and at times deemed irrelevant for major scientific debates. This creates a hierarchy of knowledge, wherein experiences, issues, and solutions originating in the South are rendered invisible or considered less legitimate - unless they are filtered, translated (even symbolically), and accepted by institutions from the Global North.

In Collective/Public Health, this dynamic has serious consequences. Successful practices and policies, such as the Brazilian Family Health Strategy, often do not receive the global visibility they deserve because they are seen as local responses to “regional” issues rather than as innovations with universal potential. The result is that knowledge, produced in dialogue with complex realities, ceases to circulate widely, while reinforcing a unidirectional model of science centered on wealthier countries and major international publishers.

Therefore, we argue that it is necessary to reconsider the meaning of internationalization in scientific publishing. Undoubtedly, journals from the Global North are internationalized - or we might say international - even when their names include “American”. In those cases, the journal is international due to its role in attracting submissions from all over the world, with scientific hegemony of those countries and the draining resources that could otherwise be applied to the production of knowledge itself.

But do we desire a single internationalization or multiple internationalizations ^6^ ^,^ ^9^? In this Editorial, we seek to problematize internationalization by understanding it as a process with distinct perspectives. Disseminating knowledge produced in one country to others, whether by publishing or incorporating it into research developed in various countries, is both desirable and necessary. We regard Science, in its many fields, as a universal public good. But for whom and how should articles on topics that are undoubtedly significant in the national scenario be disseminated to other contexts that could also benefit from this knowledge? By paying APCs? By publishing in English ^10^?

This internationalization is not a two-way process with reciprocal exchange. As new scientific hubs emerge, internationalization is no longer synonymous with engagement with the Global North. SciELO itself, a pioneer in open access, hosts journals from 15 countries (information updated on February 23, 2025), with 127 journals in the field of Health Sciences. The model of scientific publication in which neither the author nor the reader pays, known as diamond open access, is essential for democratizing science, ensuring free access for readers and authors from anywhere in the world, and should be supported by nonprofit institutions and associations, as we stated in a previous editorial ^3^.

The role of the BRICS in leading a multipolar world could be a major stimulus for communicating the science produced in peripheral countries, beyond the traditional role of being mere data collection sites. What is possible to expand the internationalization of scientific publishing in a multipolar context? Analyzing the dynamics of this process, considering both collaborative networks and the publication site, is the task set for constructing a multipolar model of scientific publishing. More than simply occupying rankings, the goal is to ensure that the knowledge produced in Brazil can circulate, be recognized, and contribute effectively to global science.

Science is the collective heritage of all peoples, cultures, and territories. Thus, the pathways to internationalization should serve to broaden access, diversity, and the relevance of knowledge - not to reproduce geopolitical or epistemological asymmetries. Not one single story ^11^, as Chimamanda Ngozi Adichie notes (see https://www.youtube.com/watch?v=D9Ihs241zeg), but a science that is not limited to a single worldview.
