# Supplementary material for: Internationalization of scientific publishing in a multipolar world
Source: Cad Saude Publica. 2025 May 23;41(5):e00067325. doi: 10.1590/0102-311XEN067325 (PMC12108106; doi:10.1590/0102-311XEN067325)
Supplement: Translation

# Translation: Internacionalização da publicação científica em um mundo multipolar

**DOI:** 10.1590/0102-311XPT067325

**Authors:** Marilia Sá Carvalho, Luciana Dias de Lima, Luciana Correia Alves

**Affiliations:** 1 Programa de Computação Científica, Fundação Oswaldo Cruz, Rio de Janeiro, Brasil.; 2 Escola Nacional de Saúde Pública Sergio Arouca, Fundação Oswaldo Cruz, Rio de Janeiro, Brasil.; 3 Instituto de Filosofia e Ciências Humanas, Universidade Estadual de Campinas, Campinas, Brasil.

A internacionalização dos periódicos brasileiros vem sendo debatida amplamente e se apresenta como um elemento importante para o fortalecimento da ciência nacional e sua integração ao debate acadêmico em todo o mundo. De forma geral, a internacionalização refere-se ao processo de inserção de revistas, pesquisadores e instituições em circuitos globais de produção, disseminação e avaliação científica. Isso envolve a colaboração entre autores de diferentes países, a presença em bases de dados internacionais e o cumprimento de critérios de qualidade estabelecidos por organismos globais.

Entretanto, frequentemente apresentada como um caminho para promover visibilidade e trocas necessárias, o debate da internacionalização carrega uma série de contradições, especialmente quando observada a partir da perspectiva dos países com menor inserção nos circuitos tradicionais da ciência internacional. Não sem razão, apesar do avanço substancial do Brasil nos últimos anos, ainda é um desafio para o país posicionar suas revistas e respectivas publicações no conjunto dos principais periódicos do mundo.

Em uma vertente mais pragmática, alguns autores apontam os entraves e as estratégias possíveis para ampliação do impacto internacional das publicações brasileiras ^1^ ^,^ ^2^. Entre as limitações, está a barreira linguística enfrentada por pesquisadores de países não anglófonos ^3^. SciELO, a grande biblioteca digital de acesso aberto que reúne periódicos científicos de diversos países, sustenta a publicação de artigos em inglês como indispensável ao aumento de citações e dos indicadores de impacto, de forma a ampliar o reconhecimento e a visibilidade das pesquisas e autores à comunidade internacional ^4^. Também a baixa diversidade geográfica da composição dos conselhos editoriais, de autores e de revisores é um aspecto que desafia as revistas brasileiras. O corpo editorial de muitas revistas é majoritariamente restrito ao país, o que limita a inserção internacional e o reconhecimento externo. Por sua vez, autores de países centrais somente publicam no Brasil artigos feitos em parceria com autores nativos ou da América Latina.

Outra vertente desse debate questiona o que se entende por internacionalização da ciência e das revistas científicas ^5^. CSP já abordou essa questão em outros fóruns ^6^, mas é bom lembrar que apenas seis conglomerados controlam o mercado editorial científico, com margens de lucro espantosas ^7^. O acesso aberto, associado à internacionalização, é viabilizado por meio de taxas cobradas dos autores, agências de financiamento e instituições. Estima-se que cientistas tenham desembolsado mais de USD 1 bilhão em quatro anos para publicar em acesso aberto nesses veículos. Essa concentração e o alto custo dificultam a inserção de periódicos e pesquisadores de países com menos recursos, comprometendo uma internacionalização mais equitativa e inclusiva.

O Portal de Periódicos CAPES (https://www.periodicos.capes.gov.br/index.php/sobre/nossa-historia.html), criado em 2000, trouxe acesso aos artigos de mais de 50 mil revistas científicas para cerca de 500 instituições brasileiras, garantindo o acesso ao conhecimento que é produzido no exterior, nos países centrais principalmente. Recentemente, a CAPES (Coordenação de Aperfeiçoamento de Pessoal de Nível Superior) assinou acordos transformativos com editoras comerciais, garantindo o pagamento das taxas de processamento de artigos (*article processing charges* - APC; https://www.periodicos.capes.gov.br/index.php/acessoaberto/acordos-transformativos.html). O objetivo é facilitar a transição do acesso por assinatura ao modelo de acesso aberto, o que se justifica por dar maior visibilidade à produção científica e tecnológica nacional. Além disso, a publicação nessas revistas deixará de ser possível somente para os pesquisadores que têm bons financiamentos de projetos, democratizando a publicação. Entretanto, a estratégia de pagar APC para publicar no exterior busca internacionalizar a ciência brasileira e, simultaneamente, transfere volumes expressivos de recursos do país para fora. Em 2025, o contrato da CAPES com a editora Wiley custou USD 8,3 milhões (dado obtido via Lei de Acesso à Informação, protocolo 23546.018379/2025-17). Em comparação, o edital CNPq/CAPES de 2024 destinou apenas BRL 6 milhões para apoiar 272 periódicos nacionais, o que corresponde a uma média de BRL 22.000 por revista.

Os acordos transformativos combinam internacionalização receptiva, na qual se lê o que está sendo produzido no exterior, e exportadora, que busca divulgar a ciência produzida localmente. Ambos os processos são inerentes a um mundo unipolar, no qual o Sul Global está, por definição, em posição subalterna ^8^. O chamado Sul Global é composto por países tão diversos como Brasil, Índia, África do Sul, México, Nigéria, Argentina, Indonésia e outros da América Latina, África e partes da Ásia. Trata-se de uma categoria geopolítica e epistemológica que abrange nações historicamente marginalizadas nos fluxos internacionais de ciência, tecnologia e inovação. Em contraposição, o Norte Global inclui países como Estados Unidos, Reino Unido, Alemanha, França, Canadá, Japão, entre outros, que concentram os principais centros de pesquisa, editam a maior parte dos periódicos de alto fator de impacto e definem os parâmetros de excelência científica em escala mundial.

Nesse contexto, a internacionalização muitas vezes se configura como uma via de mão única, marcada pela subalternidade do conhecimento produzido no Sul Global. Essa subalternidade não é apenas econômica ou tecnológica. Ela se manifesta na validação desigual de saberes, na imposição de normas de publicação, e na ideia implícita de que a ciência feita nos países centrais é universal, enquanto a produzida na periferia é local, contextual e, por vezes, irrelevante para os grandes debates científicos. Isso cria uma hierarquia de conhecimentos, na qual as experiências, problemas e soluções originadas no Sul são invisibilizadas ou consideradas menos legítimas, a menos que sejam filtradas, traduzidas (inclusive simbolicamente) e aceitas por instituições do Norte.

Na Saúde Coletiva/Saúde Pública essa dinâmica tem consequências graves. Práticas e políticas exitosas, como a Estratégia Saúde da Família, muitas vezes não ganham a devida visibilidade global porque são vistas como respostas locais a problemas “regionais”, e não como inovações com potencial de contribuição universal. O resultado é que saberes produzidos em diálogo com realidades complexas, deixam de circular amplamente, enquanto se reforça um modelo de ciência unidirecional, centrado nos países mais ricos e nas grandes editoras internacionais.

Por isso, argumentamos que é necessário rever o sentido da palavra “internacionalização” no campo da publicação científica. Certamente as revistas do Norte Global são internacionalizadas, ou podemos dizer internacionais, mesmo quando no seu nome levam a palavra *American*. Nesse caso, a revista é internacional por seu papel de concentração de submissões de artigos de todo o mundo, com hegemonia científica desses países e drenagem de recursos que poderiam ser aplicados na produção do conhecimento em si.

Mas queremos uma internacionalização ou múltiplas internacionalizações ^6^ ^,^ ^9^? Neste Editorial, procuramos problematizar a internacionalização entendendo-a como um processo com perspectivas distintas. Levar o conhecimento produzido em um país para outros, seja publicando ou incorporando-o às pesquisas desenvolvidas em diversos países, é desejável e necessário. Entendemos a ciência, nas diversas áreas do conhecimento, como um bem público universal. Mas para quem e como levar artigos sobre temas certamente relevantes no cenário nacional para outras realidades que também se beneficiariam desse conhecimento? Pagando APCs? Publicando em inglês ^10^?

Internacionalização assim, não é uma via de mão dupla, com espaço nos dois sentidos. À medida em que emergem novos polos científicos, a internacionalização não é mais sinônimo da relação com países do hemisfério Norte. O próprio SciELO, pioneiro no acesso aberto, reúne periódicos de 15 países (informação atualizada em 23 de fevereiro de 2025), com 127 revistas no campo das Ciências da Saúde. O acesso à publicação científica, no qual nem autor nem leitor pagam, denominado “acesso aberto diamante”, é essencial para a democratização da ciência, garantindo o acesso livre a leitores e autores de qualquer parte do mundo, e deve ser sustentado por instituições e associações sem finalidade lucrativa, como afirmamos em editorial anterior ^3^.

O papel dos BRICS na liderança de um mundo multipolar pode ser um grande estímulo para a comunicação da ciência produzida em países periféricos, para além da antiga participação como local de coleta de dados. O que é possível para ampliar a internacionalização da publicação científica em contexto de multipolaridade? Analisar a forma como se dá esse processo de internacionalização, tanto considerando as redes de colaboração quanto o local de publicação, é a tarefa que está colocada para construir um modelo multipolar da publicação científica. Mais do que ocupar rankings, trata-se de garantir que o conhecimento produzido no Brasil possa circular, ser reconhecido e contribuir efetivamente para a ciência global.

A ciência é um patrimônio coletivo de todos os povos, culturas e territórios. Assim, os caminhos da internacionalização devem servir para ampliar o acesso, a diversidade e a relevância do conhecimento, e não para reproduzir assimetrias geopolíticas ou epistemológicas. Nem uma única história ^11^, como fala Chimamanda Ngozi Adichie (https://www.youtube.com/watch?v=D9Ihs241zeg), nem uma ciência limitada a uma só visão de mundo.

# Translation: Internacionalización de la publicación científica en un mundo multipolar

**DOI:** 10.1590/0102-311XES067325

**Authors:** Marilia Sá Carvalho, Luciana Dias de Lima, Luciana Correia Alves

**Affiliations:** 1 Programa de Computação Científica, Fundação Oswaldo Cruz, Rio de Janeiro, Brasil.; 2 Escola Nacional de Saúde Pública Sergio Arouca, Fundação Oswaldo Cruz, Rio de Janeiro, Brasil.; 3 Instituto de Filosofia e Ciências Humanas, Universidade Estadual de Campinas, Campinas, Brasil.

La internacionalización de los periódicos brasileños ha sido debatida ampliamente y se presenta como un elemento importante para fortalecer la ciencia nacional y su integración en el debate académico a nivel mundial. En general, consiste en el proceso de inserción de periódicos, investigadores e instituciones en circuitos globales de producción, difusión y evaluación científica. Esto implica la colaboración entre autores de varios países, la presencia en bases de datos internacionales y el cumplimiento de los criterios de calidad establecidos por las agencias a nivel mundial.

Aunque la presentan como una forma de promover la visibilidad y los intercambios necesarios, el debate sobre la internacionalización conlleva una serie de contradicciones, especialmente cuando se observa desde la perspectiva de países con menor inserción en los circuitos tradicionales de la ciencia internacional. A pesar de los considerables avances de Brasil en los últimos años, el país todavía se enfrenta a un desafío para inserir sus periódicos y sus publicaciones entre los principales periódicos del mundo.

En una perspectiva más pragmática, algunos autores plantean los obstáculos y las posibles estrategias para expandir el impacto internacional de las publicaciones brasileñas ^1^ ^,^ ^2^. Entre las limitaciones se encuentran la barrera lingüística que enfrentan los investigadores de países que no hablan inglés ^3^. SciELO, la gran biblioteca digital de acceso abierto que reúne periódicos científicos de varios países, apoya la publicación de artículos en inglés como indispensable para aumentar las citas y los indicadores de impacto, con el fin de aumentar el reconocimiento y la visibilidad de la investigación y de los autores ante la comunidad internacional ^4^. Además, la baja diversidad geográfica en la composición de los consejos editoriales, de los autores y de los revisores es un aspecto desafiante para los periódicos brasileños. El consejo editorial de muchos periódicos se restringe principalmente al país, lo que limita la inserción internacional y el reconocimiento externo. A su vez, los autores de los países centrales solo publican en Brasil artículos realizados en colaboración con autores nativos o de América Latina.

Otro aspecto de este debate interroga lo que se entiende por internacionalización de la ciencia y de los periódicos científicos ^5^. CSP ya ha abordado este tema en otros foros ^6^, pero cabe recordar que solo seis conglomerados controlan el mercado editorial científico, con asombrosos márgenes de beneficio ^7^. El acceso abierto asociado a la internacionalización es posible gracias a las tarifas cobradas a los autores, a agencias de financiación y a instituciones. Se estima que los científicos han desembolsado más de mil millones de dólares en cuatro años para publicar en acceso abierto en estos medios. Esta concentración y el alto costo dificultan la inserción de periódicos e investigadores de países con menos recursos, lo cual compromete una internacionalización más equitativa e inclusiva.

El Portal de Periódicos CAPES (https://www.periodicos.capes.gov.br/index.php/sobre/nossa-historia.html) creado en 2000 permitió el acceso a artículos de más de 50.000 periódicos científicos a aproximadamente 500 instituciones brasileñas, lo cual garantiza el acceso al conocimiento que se produce en el extranjero, principalmente en los países centrales. Recientemente, CAPES (Coordinación de la Formación del Personal de Nivel Superior) ha firmado acuerdos transformadores con editoriales comerciales, lo cual garantiza el pago de los cargos por procesamiento de artículos (*article processing charges* - APC; https://www.periodicos.capes.gov.br/index.php/acessoaberto/acordos-transformativos.html). El objetivo es facilitar la transición del acceso por suscripción al modelo de acceso abierto, lo que se justifica por una mayor visibilidad a la producción científica y tecnológica nacional. Además, la publicación en estos periódicos ya no será posible solo para los investigadores que cuentan con una buena financiación de proyectos, lo cual democratiza la publicación. Sin embargo, la estrategia de pagar APC para publicar en el extranjero busca internacionalizar la ciencia brasileña y, al mismo tiempo, transferir importantes volúmenes de recursos del país al extranjero. En 2025, el contrato de CAPES con la editorial Wiley costó USD 8,3 millones (datos obtenidos de la Ley de Acceso a la Información, protocolo 23546.018379/2025-17). En comparación, la convocatoria CNPq/CAPES de 2024 asignó solo BRL 6 millones para apoyar 272 periódicos nacionales, lo que corresponde a un promedio de BRL 22.000 por periódico.

Los acuerdos transformadores combinan la internacionalización receptiva, en la que se lee lo que se produce en el extranjero, y la exportación, que busca difundir la ciencia producida localmente. Ambos procesos son inherentes a un mundo unipolar, en el que el Sur Global está, por definición, en una posición subordinada ^8^. El llamado Sur Global está compuesto por países muy diversos como Brasil, India, Sudáfrica, México, Nigeria, Argentina, Indonesia y otros en América Latina, África y partes de Asia. Es una categoría geopolítica y epistemológica que abarca las naciones históricamente marginadas en los flujos internacionales de la ciencia, tecnología e innovación. En cambio, el Norte Global incluye países como Estados Unidos, Reino Unido, Alemania, Francia, Canadá, Japón, entre otros, que concentran los principales centros de investigación, editan la mayoría de los periódicos de alto factor de impacto y definen los patrones de excelencia científica a nivel mundial.

En este contexto, la internacionalización frecuentemente se configura como una vía única marcada por la subalternidad del conocimiento producido en el Sur Global. Esa subalternidad no es solo económica o tecnológica. Se manifiesta en la validación desigual del conocimiento, en la imposición de normas para publicación y en la idea implícita de que la ciencia realizada en los países centrales es universal, mientras que la producida en la periferia es local, contextual y a veces irrelevante para los grandes debates científicos. Esto crea una jerarquía de conocimientos, en la cual las experiencias, los problemas y las soluciones que provienen del Sur se vuelven invisibles o se consideran menos legítimas a menos que sean filtradas, traducidas (incluso simbólicamente) y aceptadas por las instituciones del Norte.

En Salud Colectiva/Salud Pública, esta dinámica tiene graves consecuencias. Las prácticas y políticas exitosas, como la Estrategia de Salud Familiar de Brasil, frecuentemente no han obtenido la debida visibilidad global porque la consideran como respuestas locales a problemas “regionales”, pero no como innovaciones con potencial de contribución universal. El resultado es que el conocimiento producido en diálogo con realidades complejas, deja de circular ampliamente, mientras se refuerza un modelo científico unidireccional centrado en los países más ricos y en las principales editoriales internacionales.

Por lo tanto, argumentamos que es necesario revisar el significado de la palabra “internacionalización” en el campo de la publicación científica. Ciertamente los periódicos del Norte Global están internacionalizadas, o podemos decir internacionales, incluso cuando en su nombre llevan la palabra *American*. En este caso, el periódico es internacional por su papel en la concentración de envíos de artículos de todo el mundo, con hegemonía científica de estos países y pérdida de recursos que podrían aplicarse en la propia producción de conocimiento.

Pero ¿queremos una internacionalización o múltiples internacionalizaciones ^6^ ^,^ ^9^? En este Editorial buscamos problematizar la internacionalización entendiéndola como un proceso con diversas perspectivas. Es deseable y necesario llevar el conocimiento producido en un país a otro, ya sea mediante su publicación o incorporación a investigaciones desarrolladas en varios países. Consideramos la ciencia, en las diversas áreas del conocimiento, como un bien público universal. Pero ¿para quién y cómo llevar artículos sobre temas ciertamente relevantes en el escenario nacional a otras realidades que también se beneficiarían de este conocimiento? ¿Mediante el pago de APC? ¿O por publicaciones en inglés ^10^?

La internacionalización como esta no es una vía única, con espacio en ambas direcciones. A medida que surgen nuevos centros científicos, la internacionalización ya no es sinónimo de la relación con los países del hemisferio Norte. La propia SciELO, pionera en el acceso abierto, reúne periódicos de 15 países (información actualizada el 23 de febrero de 2025), con 127 periódicos en el ámbito de las Ciencias de la Salud. El acceso a la edición científica, en que ni el autor ni el lector pagan llamado “diamante acceso abierto”, es esencial para la democratización de la ciencia, pues garantiza el libre acceso a lectores y a autores de cualquier parte del mundo, y debe contar con el apoyo de instituciones y asociaciones sin ánimo de lucro, como afirmamos en un editorial anterior ^3^.

El papel de los BRICS en el liderazgo de un mundo multipolar puede ser un gran estímulo para la comunicación de la ciencia producida en los países periféricos más allá de la antigua participación como lugar de recolección de datos. ¿Qué se puede hacer para ampliar la internacionalización de la publicación científica en un contexto multipolar? La evaluación de cómo se desarrolla este proceso de internacionalización, tanto considerando las redes de colaboración como el lugar de publicación, es la tarea que se plantea para la construcción de un modelo multipolar de publicación científica. Más que ocupar rankings, se busca asegurar que el conocimiento producido en Brasil pueda circular, ser reconocido y contribuir efectivamente a la ciencia global.

La ciencia es un patrimonio colectivo de todos los pueblos, culturas y territorios. Así, los caminos de la internacionalización deben servir para ampliar el acceso, la diversidad y la relevancia del conocimiento, y no para reproducir asimetrías geopolíticas o epistemológicas. Ni una sola historia ^11^, como dice Chimamanda Ngozi Adichie (https://www.youtube.com/watch?v=D9Ihs241zeg), ni una ciencia limitada a una sola cosmovisión.
